# Community dialogues for child health: results from a qualitative process evaluation in three countries

**DOI:** 10.1186/s41043-017-0106-0

**Published:** 2017-06-05

**Authors:** Sandrine Martin, Jordana Leitão, Denis Muhangi, Anthony Nuwa, Dieterio Magul, Helen Counihan

**Affiliations:** 1 Malaria Consortium Mozambique, Rua Joseph Ki-Zerbo 191, PO Box 3655, Coop, Maputo Mozambique; 2 Independant consultant, Rua Custódio Bento de Azevedo n° 24, Luanda, Angola; 3 Department of Social Work and Social Administration, Makerere University, P.O.Box 7062, Kampala, Uganda; 4 Malaria Consortium Uganda, Plot 25 Upper Naguru East Road, P.O.Box 8045, Kampala, Uganda; 5 Malaria Consortium Mozambique, Av. Prof. De Moçambique, 62, Inhambane, Mozambique; 6 Malaria Consortium Headquarters, Development House 56-64 Leonard Street, London, EC2A 4LT UK

**Keywords:** Community engagement, Process evaluation, Behaviour change, Health communication, Dialogue, Zambia, Mozambique, Uganda

## Abstract

**Background:**

Across the developing world, countries are increasingly adopting the integrated community case management of childhood illnesses (iCCM) strategy in efforts to reduce child mortality. This intervention’s effectiveness is dependent on community adoption and changes in care-seeking practices. We assessed the implementation process of a theory-driven community dialogue (CD) intervention specifically designed to strengthen the support and uptake of the newly introduced iCCM services and related behaviours in three African countries.

**Methods:**

A qualitative process evaluation methodology was chosen and used secondary project data and primary data collected in two districts of each of the three countries, in purposefully sampled communities. The final data set included 67 focus group discussions and 57 key informant interviews, totalling 642 respondents, including caregivers, CD facilitators community leaders, and trainers. Thematic analysis of the data followed the ‘Framework Approach’ utilising both a deduction and induction process.

**Results:**

Results show that CDs contribute to triggering community uptake of and support for iCCM services through filling health information gaps and building cooperation within communities. We found it to be an effective approach for addressing social norms around child care practices. This approach was embraced by communities for its flexibility and value in planning individual and collective change.

**Conclusions:**

Regular CDs can contribute to the formation of new habits, particularly in relation to seeking timely care in case of child sickness. This study also confirms the value of process evaluation to unwrap the mechanisms of community mobilisation approaches in context and provides key insights for improving the CD approach.

**Electronic supplementary material:**

The online version of this article (doi:10.1186/s41043-017-0106-0) contains supplementary material, which is available to authorized users.

## Background

Low- and middle-income countries are increasingly adopting community-based strategies for delivery of basic health care, such as integrated community case management (iCCM) which aims to reduce mortality among children under 5 years. iCCM is a community-based child survival approach that provides life-saving treatment to sick children under five, for malaria, pneumonia, and/or diarrhoea, delivered by community members commonly known as community health workers (CHWs) as per the World Health Organisation’s definition [1]. Although the provision of iCCM has been shown to increase care-seeking in some contexts, just having community-based healthcare services in place is not always enough to drive appropriate uptake by local populations [2]. It is recognised that behaviour change communication and community mobilisation strategies tailored to local contexts are needed to improve uptake of iCCM at community level [3] and that specific demand-focused interventions need to be developed within programmes [4]. For iCCM programmes to be successful, communication and social mobilisation is now considered to be one of the eight components that should be included, along with coordination, costing, human resources, supply chain, service delivery, supervision, and monitoring and evaluation [5]. However, only a few studies have looked specifically at the demand-side of the iCCM strategy, identifying potential barriers and successful interventions to address these [6].

There is a general consensus that health programmes should develop specific strategies to raise awareness, encourage dialogue and involve communities in designing solutions [7]. Effectiveness trials and systematic reviews demonstrated the health impacts of interventions that supported community empowerment in low- and middle-income countries [8, 9], in particular through women’s groups [10, 11], village health committees [12], community led total sanitation [13], and other forms of community accountability [14, 15]. These have drawn attention to the essential role played by communities in improving health practices and access to services [16]. However, as noted by George AS et al., evaluations tend to focus on health outcomes. Several reviews noted that the evidence is still scarce on what does or does not work, calling for more experimental designs [17], process evaluations [18] and qualitative research [19] to clarify how effectively community participation contributes to health outcomes.

The literature recommends that future research particularly focusing on process is needed ‘to fully unlock the potential that community mobilisation approaches have to improve health and reduce mortality’ [9]. Rifkin et al. also support the need to better understand ‘what works, for whom and why?’ and recommend that process evaluations should specifically look at the views, experiences and perceptions of those involved in the programme [20]. Draper highlights that a recurrent gap in published evaluations of the effectiveness of community participation in improving health outcomes, particularly in low-income countries, is that they ‘lack detail of the community participation component’ which is essential to relate its processes to outcomes. This requires adequate description of the community context and processes leading to change [7] and the processes of adoption or rejection of a specific approach by potential users [21].

In this paper, we aim at describing and assessing a community engagement strategy, the community dialogue (CD) approach, specifically designed and implemented to raise communities and caregivers’ demand for, utilisation of, and support to the newly introduced iCCM services in three African countries.

The term ‘community dialogue’ has been used in various interventions to describe an interactive participatory communication process of sharing information between people or groups of people aimed at reaching a common understanding and consensus to address specific issues. Community dialogue approaches are inspired by the work of Paulo Freire, who postulated that dialogue provides opportunities for critical thinking, questioning of assumptions, and developing a new vision among group participants [22]. Community dialogues share with other participatory learning and action approaches the principles of respect for the community’s capacity to address its own problems, seeking local knowledge and diversity, and a set of processes that enable analysis, sharing and empowerment [23].

There is a growing body of literature on approaches using participatory learning and action in reproductive health [24], maternal and child health [25], community-led sanitation (CLTS) [13] and community conversations around gender and HIV issues HIV prevention efforts [26, 27].

In the grey literature, there are reports of a range of community dialogue operational models, mainly in the fields of HIV prevention [28], immunisation and malaria [29]. These models vary greatly in terms of process, facilitators, participants, duration, frequency and topics, depending on the specific resources and context of projects and countries where the approach is implemented. In the peer-reviewed academic literature, there is very little systematic description of the methodology and results of community dialogues in low- and middle-income countries.

**Fig. 1:**
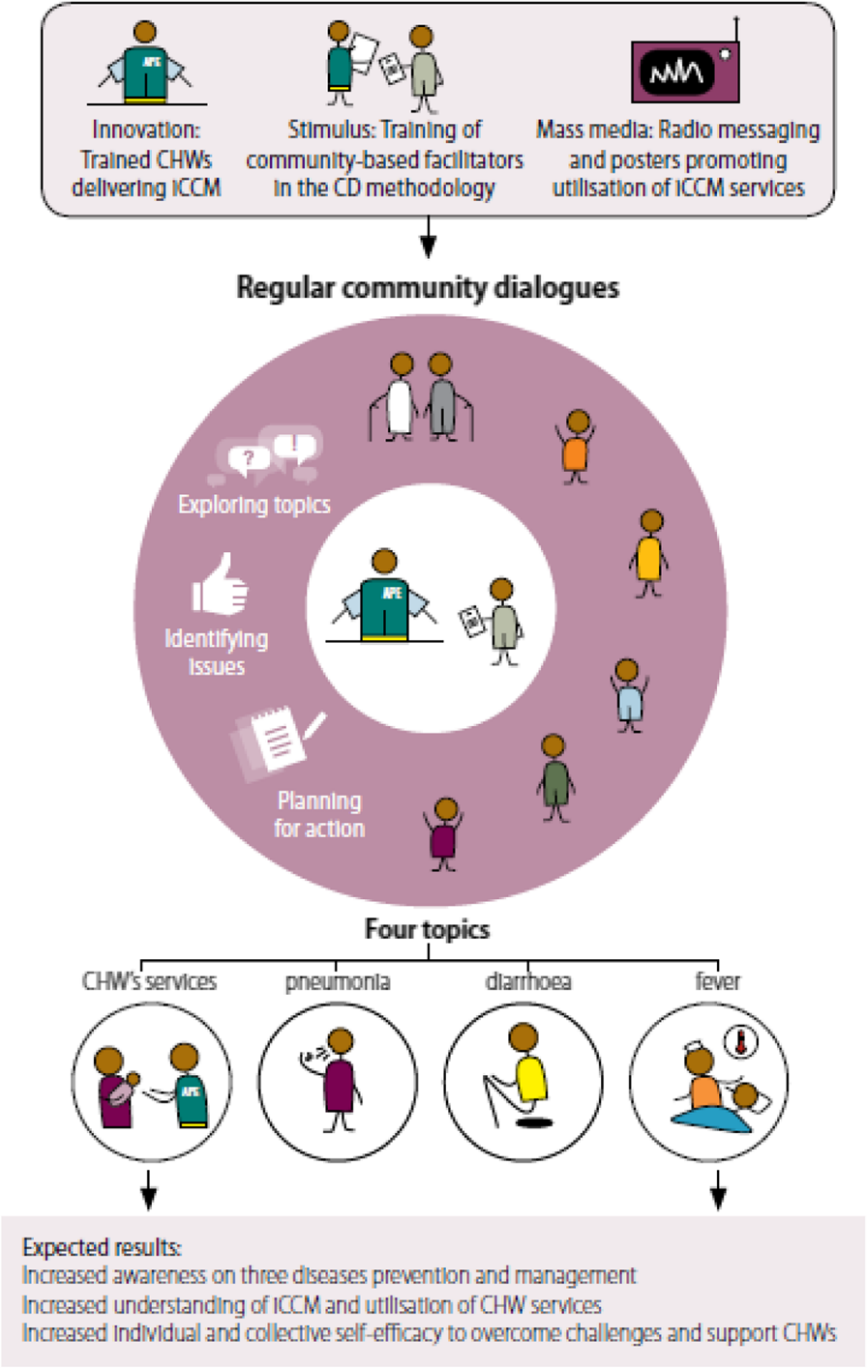
Community dialogue conceptual framework

We developed a specific community dialogue operational model, described in details elsewhere [30, 31], tailored to the context of iCCM programme as implemented by local health authorities in low-resource settings. We aimed to develop a simple and flexible CD model which could be eventually adopted by national Ministries of Health for scale up within national iCCM programmes. To this end, the model included easy-to-use tools and features which would not require skilled external facilitation for implementation and monitoring. As postulated in the theory-driven conceptual framework [see Fig. 1], which is based on the Integrative Model of Communication for Social Change [32], the overall objective of CDs is to contribute to triggering individual and social change in communities for prevention as well as timely and optimal management of childhood diseases through iCCM services. In this CD approach, CHWs and community leaders receive a 2-day training to organise and lead regular participatory CDs, without external facilitation or incentives. They use a repeatable 10-step process, around four core topics: the new child health community-based services provided by CHWs and each of the three major childhood illnesses (malaria, pneumonia and diarrhoea). During each session, community members explore a topic, identify and prioritise specific issues, and collectively agree on actions and mechanisms for the community to resolve these, within their own means and strengths. Community-based CD facilitators, usually CHWs and community leaders, are provided with a toolkit comprising of a guidebook that includes four core topic guides and a set of visual tools. These are tailored to the context of each country to support the participatory discussions around the new community-based services and the prevention and management of three childhood illnesses, as detailed in Table 1.

**Table 1:** Adaptations of the CD model to countries’ iCCM programme features

Country	CD facilitators’ profiles	CD toolkit contents	iCCM programme features
Uganda	CHW (called Village Health Team, VHT, members) and local chairmen level 1	CD guide book (English only) and 4 thematic visual flipcharts (produced in local languages): 1 on VHT services, 1 about malaria, 1 about diarrhoea, 1 about pneumonia prevention and management. Available at http://ccmcentral.com/documents/enjoy-life-uganda-flipchart/ and http://www.thehealthcompass.org/project-examples/enjoy-life-community-dialogue-booklet	CHWs are volunteers who do not receive any financial incentive. They receive a 5-day training. They provide iCCM services (malaria, pneumonia and diarrhoea) including newborn care counselling. As per national guidelines, they are not trained nor tasked with organising regular health promotion activities. 2 CHWs are trained for each village.
Mozambique	CHWs (locally referred to as APE, *Agentes Polivalentes Elementares*, in Portuguese) and the local first level administrative leader of each CHW catchment area.	CD guide book and set of 18 pictorial flash cards (Portuguese only). Available at http://ccmcentral.com/documents/cartas-para-saude-programa-dos-apes-mozambique/	CHWs are semi-volunteers who receive a monthly stipend of about USD 35. They receive a 4-month training. They provide both curative and preventive care: Curative services: management of malaria for all age groups, pneumonia and diarrhoea for children under five, first aid. Preventive services: Health promotion and counselling through home visits and group meetings. They usually cover large catchment areas.
Zambia	Members of community-based organisations (Neighbourhood Health Committees, Safe Motherhood Action groups), community leaders (traditional, religious, elders, traditional birth attendants) and CHWs	CD guide book cards, interactive poster and flash cards (Bemba and English languages). Available at http://www.thehealthcompass.org/project-examples/child-health-interactive-poster-zambia and http://ccmcentral.com/wp-content/uploads/2014/04/Community-Dialogues-for-Healthy-Children-Zambia_MOH-Zambia-MC_2012.pdf	CHWs are volunteers who do not receive any financial incentive. They receive a 5-day training. They provide iCCM services (malaria, pneumonia and diarrhoea). As per national guidelines, they are not trained nor tasked with organising regular health promotion activities. They usually cover large catchment areas.

This CD model differs from a community dialogue intervention described by Figueroa [33] in that facilitators did not receive any stipend for conducting dialogues and the duration and frequency of the dialogues were not pre-established by the project. Our community dialogue approach is similar to community conversations insofar as both approaches ‘have an explicit ‘problem solving’ agenda, aiming to spur critical thought that enables people to formulate local solutions to local issues’ as put by Campbell [27]. Unlike Campbell’s model, the CD facilitators in our approach were not outside facilitators but members of the very communities were the dialogues were held. This CD approach also differs from social accountability approaches such as community score cards [34] in that it did not aim to address directly the services provided at health facility referral level, but only CHW’s services.

As in the CLTS approach, the CD approach aims to influence individual practices through igniting community-wide behaviour change and collective action through addressing subjective and social norms around health practices [35]. However, the CD approach is not prescriptive allowing communities to identify their own issues and course of actions.

The CD approach resonates with participatory action research (PAR) defined by Chambers as ‘an approach to enable local people to share, enhance and analyse their knowledge of life and conditions, to plan and to act [36].’ It differs from PAR insofar that the intervention was not implemented as part of a research project; it was developed by Malaria Consortium to be integrated into the delivery of iCCM by health authorities, as a key strategy for uptake of iCCM services.

The CD approach was introduced by local health authorities in 2012 in underserved rural areas of Uganda, Zambia, and Mozambique within the context of progressive roll-out of iCCM implementation from 2010, supported by Malaria Consortium.

The CD intervention targeted the project areas, that is about 150–300 communities, each one defined as a CHW catchment area, in Mozambique (Inhambane province, 7 districts, population size about 500,000), Uganda (8 districts of the Mid-Western region, population size about 2.2 million) and Zambia (all 7 districts of Luapula province, population size about 830,000). In Uganda and Zambia, the CHWs were community members who provided their services as volunteers and received 6 days training in iCCM, while in Mozambique the CHWs were also community members but received a small stipend and were given 4-month training which included iCCM.

After 1 year of implementation, a process evaluation was conducted to assess communities’ response to the CD approach, in terms of outreach, relevance and intermediate results.

## Methods

We used a qualitative process evaluation method [37] to look at the process of adaptation of this approach by local community-based facilitators and participants in purposefully sampled communities, to capture the views and perceptions of those primarily involved in the CD approach and to identify the features of successful and unsuccessful adoption in each context [19]. In order to assess the actual implementation of the intervention and how that could affect desired outcomes, the six key components of a process evaluation [37] were explored qualitatively: fidelity (the extent to which the intervention was delivered as planned), dose delivered (the amount of CDs delivered and the quality of these), dose received (the extent to which participants actively engage with and value the intervention), reach (the proportion of the target audience that participates in the CD intervention), recruitment (the procedures used to approach and attract participants and their relevance) and context (other aspects of the larger social, political and economic environment that may influence intervention implementation). For each evaluation component, specific qualitative evaluation questions were developed, and both secondary and primary data sources identified. Data sources’ selection aimed at capturing the varied perspectives and point of views of the participants in the intervention. These included community members receiving the intervention, CHWs, community leaders and CD facilitators delivering the intervention, as well as CD trainers and project staff supporting the intervention’s delivery. The process evaluation questions and triangulated data sources are provided in Additional file 1.

Secondary data sources included project design and monitoring documents, including training reports, CD observation reports and a sample of CD monitoring sheets collected from the community dialogue facilitators. Primary data were collected using focus group discussions (FGDs) and key informant interviews (KIIs), in 20 purposively sampled communities across the three countries. The sample size was considered a priori appropriate for enabling deep case-orientated enquiry across target groups while also considering the limitations of time and financial resources available.

In line with the objective to assess the efficiency of the model after 1 year of implementation, two districts were selected in each country, based on a single criterion: that they had received CD trainings approximately a year or more before data collection (in June 2012). We considered that other districts, where the intervention was introduced later, might not have had enough implementation experience to generate meaningful insights.

A total of 20 communities (8 each in Mozambique and Zambia, 4 in Uganda) were thus purposefully sampled. A smaller number of communities were selected in Uganda compared to the other two countries because of financial constraints. The purposeful sampling was to identify “information-rich” cases [38], defined as either communities where the CD approach was well uptaken, considered as ‘high uptake’, or communities where it was not well taken up, classified as ‘low uptake’.

Despite community engagement being a key component of iCCM programmes, the national routine monitoring and evaluation systems and tools did not systematically capture process data on the CD activities. Therefore, the sampling strategy relied on available but partial data complemented by insights from local stakeholders to identify information rich communities.

High and low uptake communities were identified by reviewing the available project’s monitoring data, mainly CD monitoring sheets filled in by CHWs and collected at the health centre level by Malaria Consortium during routine monitoring activities a year after CD training. This database did not capture all of the communities targeted by the intervention but only a sample, namely those whose CHW had filled in written monitoring sheets and delivered these to their catchment health centre. Within this sample, communities with the highest number of CDs conducted in the past 12 months were classified as ‘high uptake’ and communities with the smallest number of CDs were classified as ‘low uptake’. Among high CD uptake communities, those who had a community dialogue within the previous month were prioritised to increase the quality of participants’ recall of their CD experience and proceedings. Where several communities potentially met the selection criteria, the study sites were identified in consultation with local project staff who contributed insights and local knowledge.

Study participants included male and female caregivers whom the intervention intended to benefit and included caregivers who attended CDs at least once and caregivers who did not attend any: CD facilitators–CHWs and community leaders (CLs)–other community influential members, and CD facilitator trainers who were mainly district-level health staff. KIIs or FGDs were selected as appropriate for each target group; during field work, when the group targeted for FGD did not total a minimum of six participants, the field teams were instructed to conduct individual interviews instead of FGDs. The final data set included 67 FGDs and 57 KIIs, across the three countries, totalling 642 respondents, as detailed in Table 2. The research team considered that data saturation has been reached.

**Table 2:** Data collection methods and number of respondents by country

Country	Study sites	Data collection period	Number of FGDs	Number of respondents in FGDs	Number of KIIs	Number of respondents in KIIs	Total Number of respondents
Mozambique	Inhambane province, Inhassoro and Govuro districts	Oct 13	29	211	38	38	249
Breakdown of respondents by target group	CD facilitators (CHWs/CLS)		2	12	22	22	34
	Caregivers non participants in CD		10	78	1	1	79
	Caregivers CD participants		10	60	5	5	65
	Influential members		7	61	6	6	67
	CD trainers		0	0	4	4	4
Uganda	Mid-West region, Kyankwanzi and Buliisa districts	Nov 13	19	168	6	6	174
Breakdown of respondents by target group	CD facilitators (CHWs/CLS)		4	36	4	4	40
	Caregivers non participants in CD		4	44	0	0	44
	Caregivers CD participants		8	64	0	0	64
	Influential members		3	24	0	0	24
	CD trainers		0	0	2	2	2
Zambia	Luapula province, Kawambwa and Milenge districts	Sep-13	22	206	13	13	219
Breakdown of respondents by target group	CD facilitators (CHWs/CLS)		6	36	9	9	45
	Caregivers non participants in CD		8	87	0	0	87
	Caregivers CD participants		8	83	0	0	83
	Influential members		0	0	0	0	0
	CD trainers		0	0	4	0	0
Grand total			70	585	57	57	642

Recruitment of study participants was done a day before the interviews and FGDs. Influential leaders were identified in consultation with the village heads and the CHWs. Participants and non-participants in CDs were identified by a ‘random walk’ method using a varying skip pattern depending on village size. This involved field researchers starting their walk from a central point in the village, each following a different direction in order to cover various areas of the community and selecting a house on average every five houses; they then checked for potential respondents in the household by asking for caregivers of under-five children and by asking this person if she/he had ever participated in a community dialogue; when a CD participant was identified, field researchers also probed for the approximate date of latest dialogue attended. This process was repeated until the required number of participants was reached.

The study was approved by the Humanities and Social Sciences Research Ethics Board of the University of Zambia, IRB 00006464, and by the Uganda National Council for Science and Technology (UNCST) as a component of the evaluation of the PIONEER and CIDA-ICCM projects, under reference HS 666. In Mozambique, this study was approved by the Inhambane Province Health Directorate. The teams were trained in ethical conduct during fieldwork, and all necessary measures were followed to protect the physical, social and psychological well-being of the study participants. All respondents were informed of the objective of the study, the voluntary nature of participation and confidentiality measures. An information sheet about the aims of the study was shared with the participants in order to obtain their informed consent.

For each country, a detailed protocol and data collection guideline were developed. Specific topic guides were developed for each target group based on the information needs identified in the evaluation questions matrix, adapted and piloted in each country. Skilled field research teams were assembled and given a 3-day training covering the aims and objectives of the study. This training also included essentials of qualitative research, such as study participants’ recruitment procedures, quality data collection, notes-taking and interview techniques.

Field work for data collection was conducted in September 2013 in Luapula province, Zambia, in October 2013 in Inhambane province, Mozambique and in November 2013 in Mid-Western region, Uganda. All KIIs and FGDs were conducted in local languages and audio-recorded after obtaining informed consent from participants. The study adopted a ‘fair notes’ approach to note taking, whereby research assistants took notes during the interviews and wrote up a description of the interview content, using both notes and tape recording, preferably the same or following day in order to enhance data validity. Transcriptions were typed verbatim in English (Uganda, Zambia) and Portuguese (Mozambique) and cross-checked by field supervisors for translation accuracy and completeness as well as to enable the point of data saturation to be more effectively determined in the field. Thematic analysis of the qualitative data followed the ‘Framework Approach’ in order to provide a rich thematic description of all data collected across the different data collection methods, using a process of modified analytic induction [39]. This approach utilises pre-existing understandings or definitions (deduction), as captured in the process evaluation questions, against the data generated through the focus group discussions and interviews (induction). The aim is to most accurately represent the meaning of their experience for the target group, starting with an existing frame of reference (i.e. evaluation questions) and expanding it to accommodate unexpected data. This systematic approach to the data analysis included the following five stages: (i) familiarisation––where all transcripts were read and key emergent themes identified, (ii) development of a thematic coding frame which began with the anticipated themes under each of the six evaluation components (fidelity, dose delivered, dose received, reach, recruitment and context) and was iteratively modified to reflect themes emerging from the field data, (iii) indexing the data by cutting transcripts and re-organising excerpts into both the anticipated and generated themes, (iv) charting where thematic areas were distilled into summaries, (v) mapping and interpretation where each thematic area was analysed and contextualised.

The final stage of interpretive analysis reflected on how actual CD implementation, proceedings and outcomes matched the model described in the initial CD conceptual framework [see Fig. 1].

## Results

The results presented here focus only on the most striking points emerging from the interpretive analysis phase. Results outline how the CD model was actually implemented (extent of implementation, organisation and coordination of CDs); if it effectively allowed participants to explore topics, identify issues and make action-oriented commitments towards solving child health-related problems in the community (application of the participatory principles, dialogue proceedings and outcomes); if CDs may contribute, or not, to the expected results such as increased awareness, understanding and self-efficacy in appropriate management of childhood diseases, from the perspective of community members (perceived individual and collective changes); and what are the challenges and success factors which could inform further programme improvements (perceived strengths and weaknesses of the approach).

### Extent of implementation of the CDs

The CD model recommends a minimum of one session every quarter per CHW catchment area over a year-long period, in order to cover the four proposed discussion topics. In all countries, facilitators conducted on average one to two CD sessions every month. Diarrhoea and malaria were the most often discussed topics, while pneumonia appeared to be rarely addressed. Although the CHWs’ services topic was not frequently registered as a stand-alone topic for dialogues, they were routinely addressed through discussions about illness management. Similarly, it was reported in Mozambique and Zambia that frequently more than one topic was discussed in one session.

Besides the four core discussion topics proposed to the facilitators, the CD platform has been used to also address a wide range of issues of interest to the community, such as HIV/AIDS, tuberculosis, vaccinations, newborn and child care, rheumatism, ocular trauma, paralysis, tetanus, asthma, family planning, elderly care and control of fires for trash or bush clearing.

Topic selection was usually done either by deliberation between facilitators, based on the CD guide and also on issues that arose from the previous sessions or through open consultation with the CD participants at the beginning of the dialogue sessions to ensure relevance of the topics to the community’s concerns. In Mozambique and Uganda, a few communities organised a surveying of people’s concerns prior to holding the CDs, in one case by going door to door and in the second by asking participants gathered just prior to the CD session to select the most relevant theme. In Mozambique, it appeared that CLs were keen on using the CD platform to discuss other topics, unrelated to health but felt of importance to the community life.

Across the three countries, the average number of participants per dialogue was 35 but this number ranged widely from a dozen to over a hundred participants in some exceptional cases, indicating a tendency to conduct CDs with large groups. Most dialogues appeared to be conducted with a mixed group of male and female caregivers of children of various age groups; however, there was consistently higher attendance of women than men. Respondents in Mozambique noted that although men participants were fewer, they ended up participating more and dominating the discussions, whereas women tended to be more passive participants in the dialogues.

In Uganda and Zambia, in some communities with a history of other programmes giving incentives to secure people’s participation in community meetings, respondents felt that the lack of financial incentives did not make attendance of CDs attractive enough, especially among men.

### Organisation and coordination of community dialogues

CDs were meant to be co-facilitated by CHWs and CLs.

Facilitators in Zambia reported that they often teamed up as a group of trained facilitators for a whole catchment area and jointly organised a series of dialogues to cover the various population settlements in their zone. As a result, they were able to secure quite often the participation of health workers and other CHWs in the dialogues. This allowed discussion of more difficult technical aspects, but meant that the dialogues were implemented at the health centre, or nearby, which reduced proximity of the dialogue to people’s homes, according to respondents.

In Mozambique and Uganda, facilitators planned and organised the sessions in liaison with the local community leaders. The CLs appeared essential in mobilising community members to attend CDs.

*“I, as a CHW, alone in the community cannot make it if the structure is not organized. Without the CL it is very difficult to have the community close to us.”* (CHW, Mozambique)

In some communities, respondents’ accounts indicated that the fact that CLs considered community dialogues as a CHW’s responsibility was a contributing factor to the weak involvement of some CLs in the dialogues. In Mozambique, this was compounded by the CHWs receiving financial subsidies from the health sector for providing iCCM and health promotion services, whereas CLs do not receive any incentive for supporting this health programme.

In most cases, as stipulated in the CD model, CLs acted as chairpersons, opening the sessions and introducing the topic, while CHWs facilitated the discussions and answered technical questions that community members raised during sessions.

*“Our roles are clear although not fixed. Whenever there is a CL like the headman, they are usually asked to chair. Then we have CHWs and other people belonging to other health groups like SMAG (Safe Motherhood Action Groups), NHCs (Neighbourhood Health Committees). We all work together, and there is no discrimination. Our role is to facilitate… What we do is to ensure that the CHWs in the catchment area concerned are as active as possible. So, there is not much of the difference when it comes to organising and facilitating these meetings. We always work as a team.”* (CD facilitator, Zambia)

Some CHWs in Uganda reported they did not consider the CLs to have adequate facilitation skills and they preferred to keep them in a peripheral role. In Mozambique, some district level trainers noted that other influential members within the community leadership structure should also be involved and trained, such as traditional healers, religious leaders, and other community figures, as they were likely to have more moral influence and communication and facilitation skills than the first-level administrative leaders.

In communities where CLs’ commitment was low, CHW facilitators reported difficulties in getting people to attend the dialogues and thus resorted to using home visits or the space of other meetings, such as church, savings groups or spontaneous gatherings, as platforms for the CD sessions. In such instances, due to time constraints and setting, the facilitators were not able to conduct participatory discussions with the community members, as intended in the CD model, but only to transmit basic information in the format of a health talk.

*“I have held most of my dialogues from churches because in my community people are very difficult to mobilize. Telling people to come for a community meeting, like in the past days has been harvesting time it is almost impossible. That is why I decided to go to church because there, they are so many people. (…) I go and attend the service and after it I tell them that please do not go we have some issues we want to discuss. Since they are already at church they accept easily.”* (CHW, Uganda)

### Application of the participatory principles

The CD methodology aims at generating participatory discussions, learning and action. Overall, most respondents in the three countries highlighted the participatory nature of the CD approach, describing it as an open space to share experiences, concerns and doubts, even referring to CDs as ‘informal family meetings’ (Zambia).

*“The opening remarks are always that we should be free to participate because we are all there to learn and teach each other. So, it’s not like we sit in a classroom and they teach us. We talk about real problems in the community. These are things everyone experiences almost on a daily basis. So, we all participate.”* (CD participant, Zambia)

There were variations however in the extent to which the participatory principle was applied by facilitators in different communities, and the characteristics of the visual materials seemed to be a determining factor. CD participants and facilitators in all countries appreciated the set of visual materials provided by the project. They noted that it assisted participants, whose literacy and health literacy are usually very low, to visualise and better grasp information and that it added validity and credibility to the health information shared.

*“They (the materials) are good, because if we talk without showing the images, people will think we are lying but they believe the posters. The posters show that what the CHW says is true.”* (CHW, Mozambique)

All dialogues were conducted using the relevant local language(s) which was essential for community participants to express themselves freely; however, not all materials were available in these local languages. In Zambia, facilitators noted that the materials in the local language facilitated greatly their use of the topic guides and key messages, as they did not need to translate on the spot from English to their local language. In Uganda, some facilitators expressed challenges with the CD materials in English and recommended that future materials be produced in local languages.

In each country, the format of the visual materials influenced the way the discussions were run. In Uganda, where facilitators were provided with topical visual flipcharts, most CDs appeared to be conducted as questions and answers sessions, allowing some degree of discussion and debate.

*“He [the facilitator] was asking us questions and then we answer and we also ask questions and he answers us (…). This gave an opportunity to everyone to have a chance to talk.”* (CD participant, Uganda)

In Zambia, where facilitators were equipped with an interactive poster and a set of flash cards to be played out by participants, it was found that facilitators managed to conduct participatory sessions in most instances, through encouraging sharing of testimonies among participants, and that decisions or recommendations would come more often from the participants themselves than solely suggested by the facilitators.

*“They are always very interactive and consultative. It’s not like the facilitators come to feed us with information. They like to use a participatory approach where they want us to talk more and they just lead the discussions. Sometimes you would even forget they are there because we just start talking among ourselves and they just came in when it’s necessary.”* (CD participant, Zambia)

### Dialogue proceedings and outcomes

In the three countries, facilitators could recall and describe the 10-step methodology and considered it useful, simple and easy to apply. In practice, they applied these steps with flexibility, with significant variations across communities, and some facilitators reported making adjustments to fit their context.

*“We try to make our dialogues as logical and culturally relevant as possible within the methodology guidelines.”* (CHW, Zambia)

Overall, during the dialogues, facilitators gave specific attention to following the three stages of exploring the topic, identifying issues and action planning.

*“Overall, we saw in some of the CD sessions that we observed that although the facilitators may not be adhering strictly to the 10 steps, they were doing the main key things and in a good way to conduct the dialogues.”* (CD Trainer, Mozambique)

Exploring a topic and discussing issues were the two steps that tended to occupy a significant portion of the sessions, which contributed to filling knowledge gaps.

In Uganda, respondents recalled that specific misconceptions were identified and addressed during the dialogues, such as whether the insecticide in the mosquito nets would cause any harm to people.

“…*you see when people get new nets, some leave them outside their houses for a whole week so as the drug to reduce and yet others were discouraging community members that they should not sleep under the treated nets because they are very bad to their health. ‘If the drug can kill mosquitoes, how about you a human being?’ They would ask. So in the CDs we would try to look at these*.” (CHW, Uganda)

Community respondents in Zambia demonstrated good knowledge levels on the causes and prevention methods of malaria and diarrhoea, the benefits of seeking medical care early and the services provided by CHWs.

*“A lot of issues were identified. When we talk about things that have changed, that is where it all started from. When you look at witchcraft vs. diseases; or traditional medicine vs. modern medicine; going to the witchdoctor vs. going to the clinic; all these issues were identified during the exploration step in various meetings. Then from there, we guide the meeting through the discussion until we come to a point where all the misconceptions and information gaps are resolved.”* (CHW, Zambia)

In the three countries, CDs have led to many individual and collective action points being identified and implemented. Hygiene measures were among the most popular action points cited and implemented in Mozambique and Uganda. In Uganda, a community dialogue led to the mobilisation of community resources to fence off a borehole that was being spoiled by animals. In Mozambique, hygiene decisions ranged from personal level hygiene, such as bathing children and keeping them clean and handwashing with soap or ashes, to cleaning of public spaces and construction of latrines, pits for waste disposal and boreholes.

*“…after the discussion about prevention, we agreed on a day to do all that we agreed upon like the construction of pit latrines and digging of pits for the rubbish. We also reached the conclusion that there was poor hygiene near the boreholes that was causing much illness in the area because we were taking water from a dirty place. In the next morning, we complied with everything we had agreed upon, we cleaned the boreholes and contributed with 50 MT (USD 1.5) to fix the borehole of our neighbours.”* (CD participant, Mozambique)

Care-seeking in case of illness was another frequent subject for action planning. iCCM services being relatively newly introduced in these countries, early care-seeking at the CHW point of care was not a well-established behaviour, but appeared to gain strong momentum through the CDs, not only just for childhood diseases but also for other health conditions prevalent in the community. As an example, in a community of Mozambique, a deal was brokered with traditional healers to notify the CHW about any suspected case of tuberculosis, for the patient to be referred to the health centre.

In Zambia and Mozambique, most action points were taken as ‘community commitments’, being transformed into community norms, thus applying not only to CD participants but also to non-participants. This was made possible by the engagement of respected community leaders to support and actively participate in the CD sessions.

*“That’s why we are happy that our CLs are involved in this, because they wield a lot of power and authority. When we agree on any action points, they are usually taken on by the traditional leadership who make them into laws or statutes that must be followed by everyone in the community, including those who do not attend the dialogues. If the headman says everyone must have a covered pit latrine, that becomes law and everyone must do it. This is why you can see the change we are talking about in the communities. It’s not just because we discuss these things, it’s action!”* (CD facilitator, Zambia)

In all countries, respondents stated there was diffusion of information and decisions taken within the CD to the wider community. In Uganda and Mozambique, in villages where participation in CDs was high, even those who never physically attended the CDs were aware of what happened in the sessions. Most communities identified specific mechanisms for actual implementation and monitoring of decisions, which varied widely among communities and included both solidarity-based approaches and coercive measures. While in some communities of Mozambique, the lack of solidarity between community members was identified as an issue, in at least three others, either volunteers were recruited to assist the elderly and others facing difficulties in implementing the decisions or some community members were selected and encouraged to provide these services as a commercial activity.

*“There was this elderly man who did not have the strength anymore to cut down a tree to have stakes for his toilet. So he asked for help and we decided that each one of us would bring two wooden stakes for the construction of his toilet.”* (Community leader, Mozambique)

In Zambia and Mozambique, many of the communities reported that neighbourhood groups or a health commission were created to verify and ensure the implementation of action points agreed during dialogue sessions.

*"During CDs, we make a plan of what we are going to do within 2 months, that could be about building latrines or cleaning in our homes or assessing people’s concerns in our community, and then at next meeting, we discuss if we complied with the plan. We have groups responsible for these tasks and they have to report about what they saw happen in the community. That’s how we do it, and we are happy because we can see that our work is on track…"* (influential member, Mozambique)

Some communities made the action points compulsory by deciding on penalties to be applied to households in case of non-compliance.

*“Whoever does not comply will be charged to pay a number of chickens, or goats, such things. Sometimes, they can also be sent to plough in a chief’s farm called ‘mulima chipuba’ – meaning ‘a place where only fools are sent to plough’- as a form of punishment. Very few people would like to be seen there.”* (CD participant, Zambia)

Facilitators were of the opinion that these measures are effective in bringing about implementation of action points and change and interviewed community members demonstrated support for these measures.

*“With these measures, people have been complying with CD action points. We agreed that people cannot give birth in their homes, they need to go to the hospital. Whoever gives birth at home should pay a penalty of one lamb and 250 MT (USD 7).”* (CHW, Mozambique)

### Perceived individual and collective changes

CDs were shown to have contributed to increased awareness and knowledge among community members on a range of health topics, and specifically on childhood illness prevention and management and services provided by the CHWs. Community members interviewed, both among those who participated in CDs and those who did not, demonstrated overall correct knowledge of the causes and prevention measures for diarrhoea and malaria. Community members who attended CDs mentioned having gained new learning through their participation in the dialogues. However, as pneumonia appeared to be rarely discussed in CDs, community members still showed little knowledge about the disease and how it should be prevented and managed.

Study participants reported using new knowledge gained to improve health practices in their homes particularly in the areas of hygiene practices and mosquito nets’ use.

“*Community dialogues are good and we have learnt many things in them about health, so when we attend them at least one learns something new that he or she can use to improve on his/her health. For example for me I did not know how to make a tippy tap* 1 *but one of our group members who had attended one of the meetings and learnt how to make it came home and taught me how to make a tip tap. He showed to me how and where to put a jerry can with water plus the soap, I did not know that you can make a hole in the soap and put there a string and now I know it well, I can also teach others. So I believe that most of us have acquired knowledge from those meetings*.” (CD Participant, Uganda)

Individual level reported changes also include changes in the management of childhood illnesses, including increased and early utilisation of health services in general and of CHW services in particular. CDs were identified by respondents as a determinant of behavioural change, among other factors, particularly in relation to early care-seeking practices from a qualified provider.

*"In the past, I did not know that community meetings can be constructive. I used to resort to traditional medicines. But now, when the child is sick, I go to the health centre, that is why I say that I learned something and also I can see that most people in the community now go to the health centre when they are sick… this is all the result of community meetings."* (CD participant, Mozambique)

Respondents explained that CDs helped dispel the misconception that childhood illnesses were caused by witchcraft, as well as exploring the consequences of delayed care-seeking, and that as a result, there has been a shift in care-seeking practices, from traditional healers to CHWs as first point of care.

In Uganda, the CDs increased not only just the visibility of CHWs but also their popularity as critical service providers in addressing childhood illnesses, positioning them not only just as drug distributors but also as a link between the community and the health centres. Similarly in Zambia, facilitators highlighted that CDs had contributed to build trust of community members in the work of CHWs, and how CHWs became the frontline workers for childhood illnesses, and sources of health advice.

*“Community members did not even believe in us, that we can treat their children. But since we started educating them about the roles of CHWs, malaria, diarrhoea, and pneumonia in children, a lot of things have changed. I think they have made a huge difference in our communities.”* (CD facilitator, Zambia)

Respondents also perceived that the changes in behaviours at home were general in the community, becoming a new social norm, and thus had contributed to a reduction in the prevalence of malaria and diarrhoea cases in their communities.

*“This meeting helped us a lot, even sickness resulting from malaria, levels of diarrhoea sickness, and many such things have declined significantly because we followed what they were teaching us. I think when you walk around our villages; community life has improved significantly in many ways. The quality of life has improved of course because when you have less illness, then people are happier.”* (Community leader, Zambia)

The perceived changes were partly attributed to the CDs but also to the availability of convenient CHW services within communities. Among the advantages of procuring the services of CHWs, caregivers highlighted their geographical proximity, availability at any time of the day, their quick service that saved time, the efficacy of treatment provided and cost-saving, as CHW services are free of charge. A particular aspect that has been decisive for caregivers is the diagnosis of cause of illness performed by CHWs that traditional medicine doesn’t provide. Some respondents also mentioned learning gained through other channels, such as interaction with health workers at the health centres and messages heard over the radio, as reinforcing factors.

### Perceived strengths and weaknesses of the approach

CD participants and facilitators expressed a high appreciation for the CD format, highlighting that it made learning easier and allowed communities to identify solutions which are relevant to their context. They felt that dialogues provided a platform through which people could access health information within their village, and get their own questions and concerns discussed. Participants expressed that they enjoyed the CD forum for interaction between members, and for sharing of ideas and experiences.

*“The difference between a CD meeting and other meetings is that in the CD, you are given time and allowed to ask questions but in other meetings you are just on the receiving side of the facilitator”.* (CD participants, Uganda)

They also considered CDs more attractive and efficient than health talks and other means of communication because they were directly involved in identifying problems and local solutions, thus considering that CD is a service ‘*of the community for the community’*.

*“The strongest point for me is that these meetings are community-based. They are organised and owned by our communities. When you look at other similar meetings organised by other organizations, they can only happen when those organizations visit us. But for MC dialogues, we are able to do this even when MC staff are not here. This is the greatest advantage.”* (CD facilitator, Zambia)

Furthermore, active participation and interaction in the meetings was facilitated because all participants and facilitators were felt at ease discussing with their neighbours, family and friends. These aspects were also the most emphasised by respondents when discussing the differences between CD and other type of sensitisation programmes.

*"I think the meetings held in the area are very important because we share ideas on building our community, learn to do good things for our community, because even if someone comes to give a lecture we will learn the same, but when we (women), the residents, dialogue and make decisions about our life, we will have more impact on the population."* (CD participant, Mozambique)

Although not part of the model, we found that the implementation of the Community Dialogue approach was boosted by availability of additional support, such as mentoring or individual follow-up to the CHWs, during individual supervision visits or iCCM programme review meetings. These events allowed CHWs to reflect and discuss, with their supervisors or their peers, any challenges faced in implementing the dialogues and how to overcome them. In some cases, facilitators felt that they lacked technical knowledge to be able to answer difficult or unexpected questions, and that health centre staff were not sufficiently available to support them. In all countries, the participation of health workers whenever they attended was found to be enriching, as these experts provided answers to difficult questions raised by participants. Most community-based facilitators considered they needed more training and refresher sessions, to provide the dialogues with new or more complete information, to be able to debate other health topics which are of interest to the community, and thus to keep the CDs attractive and useful.

It was not possible to estimate the actual reach of the dialogues from CD records. Facilitators endeavoured to cover the various settlements of their catchment area, trying to reach out to a maximum of people, but this proved challenging. In Zambia, where facilitators tended to team up to cover their health centre catchment area, they highlighted long walking distances as a major challenge to mobilisation for the CDs and also to coordination between the facilitators and with other community leaders and structures. They suggested that more CD facilitators be trained and that they be equipped with bicycles as examples of solutions to reduce their workload and overcome this issue.

Respondents’ accounts indicated that CDs tended to be inclusive of a wide range of participants, not restricted to caregivers of young children, especially in Zambia. In Uganda, categories of people who rarely attended dialogues included the youth, both boys and girls because of lack of interest, and the elderly and people with disabilities because of physical constraints.

In Zambia and Uganda, where generic health promotion tasks were not initially part of the CHWs’ tasks, facilitators reported that CD organising and conduction was sometimes competing with their normal daily subsistence activities and that some form of incentives would help sustain the approach.

## Discussion

Detailed analysis reports of each country data were shared and discussed with respective country level Ministry of Health counterparts. Preliminary findings were summarised in a learning brief [31] shared with iCCM programme stakeholders in each country so that key lessons learned could inform programme improvement.

In this process evaluation, we assessed how the approach was implemented (efficiency), and not its impact (effectiveness), which would rather respond to a summative evaluation design. This study bears certain limitations, including the limited sample size that aimed at generating insights into key evaluation questions, but does not allow for empirical generalisation. The changes in care-seeking and disease prevention practices were self-reported by the respondents. They illustrate only perceived changes from the perspective of the community respondents, but the objective verification of these changes was beyond the scope of this study. Still this qualitative evaluation is a valuable contribution to understanding how the Community Dialogue model was implemented in context, and how it contributed to increased community awareness and support for iCCM services and some essential prevention practices. It provides key insights for improved feasibility of the CD model, some of which may be applicable to other settings where provision of community-based services is relevant.

Respondents’ experiences in the three countries overall indicate that the CD approach has been embraced by both facilitators and participants for its relevance, ease of use and participatory approach. Although the 10-step process was not strictly implemented as per the initial model proposed, facilitators applied it with flexibility to fit their local contexts and means.

The fact that, in both Uganda and Mozambique, a number of CHWs have applied the methodology to other health topics illustrate the flexibility of the approach and its potential for sparking open dialogue, critical thinking and questioning of assumptions on a variety of issues. Similar to results obtained in other community participation interventions [40], communities acquired new information pertaining to a range of health topics and developed skills related to problem solving. The CD approach fills a gap in reaching out to rural communities with basic health information and providing a platform for low-literacy rural communities to reflect on how information applies to their life in context. This is consistent with George’s review [16] which highlighted that when communities perceive interventions to fit with their needs it facilitates community level support and trust. Respondents’ accounts indicate that CDs contributed to increased understanding of childhood disease causes and management, clarified the services provided by CHWs and the benefits of early care-seeking in order to build trust, factors which has been shown to be key determinants in care-seeking [41, 42]. Trust in particular has been identified as a critical factor, together with inclusion of the broader community in decision-making and transparency, that helped communities work towards a common goal [40].

In the three countries, CD has been an effective tool for identifying specific feasible actions to improve health, and most communities have defined responsibilities to implement and monitor the actions agreed upon.

Respondents discussed how the CDs, among other factors, have contributed to a shift in first point of care from traditional medicine to CHWs. This corresponds to a previous review which found that community sensitization around seeking timely and appropriate treatment led caregivers to favour CHWs over traditional healers as first point of care [43]. These findings confirm that such participatory approaches can improve dialogue and action-oriented decision-making and increase participation by communities, families and individuals, which can result in increased demand for health services and information seeking/sharing and positive changes in individual behaviours and social norms and practices [18]. A recently published study demonstrated that a community dialogue intervention was effective at producing changes in underlying social and cultural factors related to HIV in Mozambique, including gender norms and roles, stigma, knowledge and some behaviours [33].

Indeed, community dialogues, through the discussion of care-seeking options and their respective outcomes and features, allow caregivers to better understand and value CHWs’ services, which is particularly important to build trust in care providers [6].

Although the mechanisms of implementation and monitoring of actions vary widely between communities, findings confirm that the active engagement of community leadership greatly influences community members’ participation in CD sessions and in generating overall community support for putting into action recommended practices such as early use of CHW services and preventative practices [43]. Agreeing and committing in public seemed to be another key facilitator in the actual implementation of individual and collective changes, as already shown by Wight et al. [44].

The CDs have also increased the visibility and popularity of CHWs not only as health service providers but also as a link between the community and the health sector, an important link for successful community engagement [16]. This also has the potential to increase recognition and value felt by CHWs which is known to be a driver of CHW motivation for providing continued and quality services [45, 46].

This study’s results also provide insights into specific features that can improve the CD model, by carefully evaluating the implementation processes, as recommended in recent reviews on the effects of community participation and behaviour change interventions [18, 19].

Similar to what has been reported in CLTS [47], community leaders carry the most authority and influence, and their role cannot be overlooked to mobilise participants and give credit to the CDs. However, our results also indicate that they may not be always best placed to facilitate community discussions, and other influential members from the larger community leadership structure should be engaged and trained. The experience in Zambia, where CD facilitators group to conduct CDs, allowed them to mobilise other community resources such as traditional and administrative community leaders as well as youth and women groups for greater outreach. Training more CD facilitators would reduce the size of their respective catchment area, and thus their workload, and potentially increase the frequency and outreach of the dialogues.

Beyond the visualisation of a health practice or condition in pictorials and the use of local languages, the type of materials used during CD sessions influenced the level of participants’ engagement. When participants had flash cards they could play and place to illustrate their story or their thinking, as in Zambia, CDs appeared more participatory than in Uganda where the flipchart formats tended to turn the CD more into a questions and answers session.

It is usually assumed that running a dialogue requires strong facilitation skills and therefore require either skilled facilitation by actors external to the community, such as in other community dialogue models implemented in some contexts to address resistance to immunisation [48], or structured and consistent training, such as implemented in Community Conversations’ models for HIV/AIDS prevention [26]. This study however indicates that equipping visual interactive tools to low-literacy facilitators who receive only a very short training allows them to overcome some facilitation barriers and manage action-oriented but participatory discussions, including with large groups.

Findings however caution that community dialogues and similar participatory approaches, while holding great potential, are no ‘magic bullets’ and that rural communities’ intentions to put their plans into action are often hindered by difficulties in accessing basic resources and services, in low-income countries [27, 47].

This study contributes qualitative insights to some of the key research questions identified for optimising the impact of iCCM on child health [49], such as factors of iCCM utilisation and the role of families’ understanding of the diseases’ symptoms, danger signs and management options in prompt care-seeking. Behaviours are dependent on a range of individual and contextual factors [50], and further research is still needed to unpack which of the specific cognitive, motivational and social determinants are effectively impacted through the CD model. Future research is needed to continue building the evidence base and explore the potential of community mobilisation approaches, such as the CD model, for improving child health. In particular, the role of such approaches could play in improving community organisation for completion of timely referrals for severely ill children and community monitoring and local accountability mechanisms for iCCM implementation.

When facility-based health workers occasionally participated in CDs, this generated useful discussion around critical service delivery issues such as staffing and patient-provider relationships. This seems to indicate that the CD approach would benefit from complementary social accountability mechanisms at health facility level, such as community score card approaches which have shown potential to improve access and quality of referral services [34, 51], through facilitating flows of information and collaboration. Combining the CD approach with a social accountability component would require more skilled facilitation and technical support from an experienced organisation for close and frequent mentoring of trained facilitators, a critical prerequisite for ensuring gains in this process [52]. As highlighted by Mafuta et al., community health workers, who are usually better educated and health-literate than their fellow community members, could play a key role in social accountability processes [15]. The expression and effective integration of community priorities has been a common challenge in efforts to involve communities in the health system planning process [53] for improved effectiveness. Such combination of approaches could potentially provide a much-needed platform for meaningful community involvement in the health system by allowing the priorities of grassroots community to be formulated, captured and eventually acted upon.

## Conclusions

Overall, this process evaluation indicates that CD can be a powerful approach to make health promotion activities of community-based volunteers more participatory and effective in addressing social norms around child care practices and specifically triggering community uptake of and support for iCCM services through building trust and cooperation within communities. It also indicates that the CD approach can be used for other health topics that may require community engagement.

However, sustaining behaviour and social change commitments will depend, among other factors, on the continued availability and quality of community-based and referral health services to meet the demand, which are key factors in sustaining utilisation. Otherwise, the effects of such demand-side intervention would be counterproductive, undermining general trust in public health services and eventually impacting negatively on the personal reputation and lives of CHWs in their communities.

## Additional file

Additional file 1: Process evaluation components, questions and data sources matrix (DOCX 15 kb)

## Abbreviations

CD: Community dialogue
CHW: Community health worker
CL: Community leader
FGD: Focus groups discussion
iCCM: Integrated community case management
KII: Key informant interviews

## Footnotes

1 A *Tippy Tap* is a simple water conserving/hygiene device used in a number of African countries, especially as a hand washing facility next to latrines, and generally consisting of a jerry can or container hanged and combined with a foot pressing mechanism to let the water flow.
