# Supplementary material for: Community dialogues for child health: results from a qualitative process evaluation in three countries
Source: J Health Popul Nutr. 2017 Jun 5;36:29. doi: 10.1186/s41043-017-0106-0 (PMC5460475; doi:10.1186/s41043-017-0106-0)
Supplement: Additional file 1: — Process evaluation components, questions and data sources matrix (DOCX 15 kb) [file 41043_2017_106_MOESM1_ESM.docx]

Process evaluation components, questions and data sources matrix

| **Process evaluation component** | **Evaluation questions** | **Data sources** |
| --- | --- | --- |
| Fidelity | The extent to which the intervention was delivered as planned |  |
|  | To what extent was the training module (and materials distribution) delivered as planned? What sessions or training components were not delivered and why? | Training reports  KII with trainer |
|  | What additional support was given to the community-based facilitators after the training? | CD Monitoring sheets  KII with trainer  FGD with CDFs |
|  | To what extent are CBFs satisfied with the training and toolkit provided? | FGD with CDFs |
| Dose delivered (completeness) | The amount of community dialogues delivered and the quality of these |  |
|  | How many community dialogues were conducted in the health catchment area and in the CHWs' respective catchment areas? What were the average number of participants/types and topics covered? | CD monitoring sheets  FGDs with CDFs |
|  | To what extent was the 10-step process applicable in context? To what extent were materials (CD toolkit) used in CDs? | CD Observation reports  FGDs with CDFs |
|  | To what extent did the CDs reflect the participatory discussion principle? (versus didactic session) | CD Observation reports  FGD with CDFs  FGDs with CD participants |
|  | To what extent was the "explore" step completed during dialogues? Did it allow to fill in knowledge gaps and correct misconceptions? | CD Observation reports  FGD with CDFs  FGDs with CD participants |
|  | To what extent was the "identify issues" step completed during CDs? Did it allow to model behaviours? Did it allow to discuss social norms? | CD Observation reports  FGD with CDFs  FGDs with CD participants |
|  | To what extent was the "action planning" step completed during CDs? Did it allow for individual and/or collective commitments? | CD Observation reports  FGD with CDFs  FGDs with CD participants |
| Dose received (Satisfaction & exposure) | The extent to which target groups (community members, CHWs, CBFs) actively engage and are satisfied with the community dialogue intervention |  |
|  | What are the strong and weak points of CDs from facilitators and participants' perspectives? (main drivers of satisfaction and/or dis-satisfaction, and barriers/facilitators to active participation in the CD) | CD Observation reports  FGD with CDFs  FGDs with CD participants  KII with Influential members |
|  | What are the perceived changes among participants in terms of knowledge about childhood diseases'management, attitudes towards CHWs, intended behaviours in case of child sickness? What are the perceived causes of these changes? | FGDs with CD participants  KII with Influential members |
|  | To what other changes did the CD approach contribute? i.e. To what extent does the CD approach contribute to increased community ownership of the ICCM programme? | FGDs with CD participants  KII with Influential members |
| Reach (participation) | The proportion of the target audience (community members, CHWs, CBFs) that participates in the community dialogue intervention. |  |
|  | What proportion of the target audience (CHW catchment area) participated in community dialogues? | CD monitoring sheets  Health Centre data |
|  | What is the average number of sessions CD participants attended? | FGDs with CD participants |
|  | Which community members participated/did NOT in CDs? What are the main drivers/reasons for not/participating in CDs? | FGDs with CD participants  FGDs with NON CD participants  FGDs with CDFs  KII with Influential members |
|  | How many of the trained facilitators are still active after 1 year of being trained? | FGD with CDFs |
| Recruitment | The procedures used to approach and attract target groups, and their relevance. |  |
|  | Were the community-based facilitators' recruitment criteria followed? Were these criteria relevant? | KII with Trainer  KII with Influential members |
|  | What are the main motivating factors/barriers for community-based volunteers to engage in the programme? | FGD with CDFs |
|  | How were participants in the CD sessions informed and mobilized? | FGD with CDFs  FGD with CD participants/NON participants  KII with Influential members |
| Context | Context: Other aspects of the larger social, political, and economic environment that may influence intervention implementation |  |
|  | What organizational, community, social or other factors affect the intervention? | FGDs with CDFs  KII Influential members  FGDs with CD participants and non-participants |
|  | i.e size of the health centre/CHW catchment area; drug stock-out or CHW inactive; underlying unsolved conflict in the community; gender roles and issues; quality of the relationship between community and public health facility ... etc. |  |
